# Brain-structural differences underlying dialect competence in the bilingual network

**DOI:** 10.1038/s41598-026-59884-y

**Published:** 2026-07-01

**Authors:** Mathias Scharinger, Jürgen E. Schmidt, Jens Sommer, Andreas Jansen

**Affiliations:** 1 Research Group Phonetics, Institute of German Linguistics, Marburg University, Marburg, Germany; 2 Research Center Deutscher Sprachatlas, Marburg University, Marburg, Germany; 3 Center for Mind, Brain and Behavior (CMBB), Universities of Gießen and Marburg, Marburg, Germany; 4 Core-Facility Brain Imaging, Faculty of Medicine, Marburg University, Marburg, Germany; 5 Department of Psychiatry and Psychotherapy, Marburg University, Marburg, Germany

**Keywords:** Dialect competence, Structural imaging, Bilingualism, Gray matter volume, Cortical thickness, Neuroscience, Psychology, Psychology

## Abstract

Research in multilingualism has provided evidence for brain structural differences between monolinguals and bilinguals. Less is known about speakers who, apart from a standard language, also show competence in a variety (dialect) of this language (“bidialectals”). In these populations, cultural differences are minimized and language competence can be compared against a common ground. We hypothesize that bidialectals show brain-structural differences to non-bidialectals, and capitalize on one of the world’s largest dialect corpus available for German. A competent dialect group (N = 26) and a group without dialect competence (N = 23) were compared by using brain structural measures, including gray matter volume (GMV) and cortical thickness (CT). Results demonstrate a whole-brain group difference, seen for CT in right orbitofrontal cortex, and for GMV in bilateral middle temporal gyrus and bilateral insula. Notably, CT in right fusiform cortex as well as GMV in bilateral middle temporal gyrus and right insula co-varied with dialect competence differently for the two groups. The structural differences and covariations with dialect competence are discussed on the background of code switching and language control. The findings suggest that dialect competence may shape brain structure in ways similar to bilingualism.

**Supplementary Information:**

The online version contains supplementary material available at 10.1038/s41598-026-59884-y.

## Introduction

Speaking more than one language is an ability of a large proportion of the world’s population^1^. Not surprisingly, there is a wide range of research in bilingualism and multilingualism^1,2^. One core discussion in this research concerns the question of whether bilingualism shapes general or specific cognitive skills, and whether acquiring two (or more) languages in parallel or subsequently comes with cognitive advantages or disadvantages. While older research has highlighted potential disadvantages e.g., in bilingual language acquisition or memory skills^3^, more recent research rather highlights cognitive advantages^4^. However, the evidence for such advantages is rather heterogenous, afforded to many uncontrolled and sometimes unknown variables, such as cultural background, specific language competence and/or bias, onset of language acquisition, and language attitude: this leads to the view that there might not be general cognitive advantages of bilingualism^5,6^. Furthermore, linguistic variables such as similarities or differences between the respective languages in bilingual settings (regarding pronunciation, word structure, grammar, etc.) are not always considered in sufficient detail, possibly leading to heterogenous results in meta-analyses^7^.

It is surprising, then, that another frequent competence—that of speaking one or more dialects next to a (standard) language—has not yet received the same attention as bilingualism. We refer to the ability of speaking or understanding more than one variety (i.e., dialect) of a language as »bidialectism«^8,9^. Note that the related, but not identical term »diglossia« refers to a functional specialization between varieties^10^, a distinction that we do not go into more detail in this study. For this reason we prefer the term bidialectism. Language variety is used as umbrella term, subsuming different levels along a vertical dimension, reaching from the »best standard« to the »strongest dialect«^11^. Some accounts of multilingualism include bidialectism and model the competence space as one or two dimensional gradient^12^. Despite these developments, the size of the literature on bidialectism is by no means comparable to the literature on bilingualism. There is, however, an increasing amount of bidialectal studies with a focus on behavioral measures^8,13–20^. Regarding the covariation of cognitive skills and bidialectal competence, there is a similarly heterogenous picture as in bilingual research. For attentional and inhibitory control, Hsu^13^ showed that bidialectals in Mandarin Chinese performed better on cognitive tasks with intermediate and high levels of difficulty, compared to a non-bidialectal control group. Antoniou, et al.^19^ found that bidialectal Greek children performed better on working memory and inhibition tasks than a non-bidialectal control group. Furthermore, the bidialectal group did not differ from a bilingual group of English-Greek speakers. The study of Poarch, et al.^16^ demonstrated that inhibitory control covaried with language dominance (bias) in a group of Standard German and Swabian German bidialectals, albeit in an unexpected direction: Balanced bidialectals (i.e., those using both varieties to the same degree) showed less inhibitory control than those with a Swabian German bias. Wu, et al.^20^ focused on bidialectal Mandarin Chinese speakers and found that attentional deployment in resolving phonological competition was better for bidialectal than for non-bidialectals. Finally, there are several studies focusing on switching costs in selecting one or the other variety. These studies either provided evidence for a correlation between variety dominance (bias) and asymmetric switching costs or for similarities in switching costs between bilinguals and bidialectals^17,21,22^.

In contrast to these findings, several authors found no evidence for differences in executive and attentional control^8,14,15,23^ when comparing bidialectals to non-bidialectals. A possible reason for the attested variability in findings might be that behavioral measures, known to subsume many cognitive operations, are not sensitive enough to detect the underlying processes. In the last decades, more direct measures of cognitive language skills have been applied, especially brain imaging measures with high temporal and/or spatial resolution as in Electroencephalography (EEG) or Magnetic Resonance Imaging (MRI). One particular interesting feature of MRI is that the technique can either focus on functional or structural aspects in multilingual control.

In emphasizing functional aspects, previous research has established the involvement of the executive control network^24^ in bilinguals. The focus on structural aspects, on the other hand, pertains to the plasticity of the human brain and its ability to change in morphology depending on the environment and on experience^25^. To this end, structural imaging has revealed that human cognitive skills are accompanied by changes in brain morphology, based on measures of gray or white matter. For instance, Gaser and Schlaug^26^ and Gaser and Schlaug^27^ have shown that the brain structure of professional musicians (pianists) significantly differs from a matched control group of non-musicians in areas supporting motor coordination and auditive accuracy. Another group of researchers have examined phoneticians and non-phoneticians and found brain structural differences between the two groups^28^. Even more importantly, they could directly show that one of the regions also covaried with the amount of phonetic training, thereby excluding a sole trait-based explanation.

Structural brain imaging analyses with a focus on network nodes (brain regions with specific functions), rather than network connections (fiber tracts, white matter) are either based on gray matter volume (GMV) or cortical thickness (CT). GMV is an approximation of tissue density^29^. CT, on the other hand, is the distance between the inner and the outer surface of cortical structure, and can be considered a complementary measure to GMV^30^. Both measures have been used in multilingual research, where it could be shown that multilingual skills covary with both GMV and CT measures in specific brain areas^31–35^. To give some illustrative examples, Hamalainen, et al.^31^ found that early bilingualism led to higher CT in frontal and temporal parts of the language network (left pars opercularis, right superior temporal gyrus). Kepinska, et al.^36^ and Ressel, et al.^37^ provided evidence for structural changes in auditory cortex, co-varying with multilingual and bilingual experience. Ressel and colleagues showed that bilingualism (compared to monolingualism) resulted in larger gray matter volume in Heschl’s Gyrus. Della Rosa, et al.^38^ and Mechelli, et al.^39^ identified the left parietal cortex as one region scaling with language experience. Pliatsikas, et al.^32^ demonstrated that GMV in the cerebellum is increased in bilinguals, and Schug, et al.^34^ showed that bilingual children had more GMV in bilateral frontal, right inferior frontal, and right superior parietal cortices. However, the authors also showed that the direction of structural differences between bilinguals and monolinguals depends on age as well as brain region. To this end, Vaughn, et al.^35^ found that bilinguals’ cortical thickness in several cortical areas was reduced compared to a monolingual control group. At the same time, the English/non-English bilinguals revealed brain regions where cortical thickness positively correlated with competence in English. Vaughn, et al.^40^ found differences in parietal lobe GMV between bilinguals and monolinguals; however, these differences were task-dependent. Finally, the meta-analysis by Danylkiv and Krafnick^7^ shows that GMV alone may not suffice to detect difference in brain morphology and that differences between languages of bilinguals should be accounted for. These examples illustrate the heterogenous pictures regarding brain structural differences between monolinguals and bilinguals.

Structural imaging studies on dialect competence are very sparse^41,42^. One of the few studies^42^ has found no significant brain structural differences between bidialectals and non-bidialectals. However, since the authors compared native speakers of German who were exposed to Swiss German (a German variety) at early ages (< 6 years) with native speakers of French who learned German as second language at intermediate ages (> 6 years), none of the participants were actually monodialectal or monolingual. The study therefore suggests that bidialectals resemble bilinguals in brain morphology.

Regarding dialect competence measures, research within the research center »Deutscher Sprachatlas« can build upon the unique dialect corpus of German, initiated and conceptualized by Georg Wenker in the late nineteenth century and digitized within the project »Regionalsprache.de«^43^. In particular, Schmidt and colleagues have introduced a relatively straightforward dialect competence measure, based on the correspondence of observed sound-based dialect skills and expected target forms in various German-speaking regions^44,45^. Participants are asked to produce the local dialect forms of specific German words. Their productions are compared against the expected forms on the basis of the dialect corpus, on which basis an accuracy score can be derived. Since the test is based on words whose stem vowels are diphthongs (i.e., vowel combinations such as [a͡ɪ] as in English “side”), the test is referred to as »diphthong test«. To illustrate the measure, consider the standard German word for “ice” ([a͡ɪs]). If a person is competent in Alemanic (spoken in the Southwestern part of the State of Baden-Württemberg in Germany), the expected local form would be [iːs]). The required cognitive skills of a fluent and competent dialect speaker would then be (a) to be able to select the appropriate word form; (b) to map it onto the standard German form and (c) to produce and articulate it correctly. The dialect competence measure is thus readily applicable for correlational analyses with cognitive and brain structural measures. We expect that this fine-grained measure may be better suited to account for unexplained variance in previous structural brain analyses on dialect competence. We further consider the »Dynamic Restructuring Model« by Pliatsikas^46^ as adequate conceptual framework for our study. In short, the model attempts to incorporate the heterogenous findings from research in bilingualism and multilingualism by focusing on language (or dialect) experience and its effect on brain structural plasticity. Pliatsikas considers continuous structural changes in the brain as depending on language learning, switching demands, and prior experience. We interpret “experience” pertaining to both languages and their varieties (dialects), and therefore believe that his account is adequate to account for bidialectism as well. Given the importance of the switching demands in his model, we consider the diphthong test to be a particularly well-suited behavioral measure that should affect brain structural plasticity.

For this reason, we here wanted to compare two closely matched participant groups that share their native language (German), but differ regarding their dialect competence. Competent dialect speakers (N = 26) were recruited through a local dialect association in the north of Hesse (Germany). We deliberately chose an area in which base dialects (i.e., varieties which are very different from Standard German regarding pronunciation, vocabulary and grammar) are still spoken today. The target area of our recruiting is attributed to Northern Central Hessian, Northern Hessian and their respective transition zone^47^. The varieties there are characterized by the fact that substantial differences in pronunciation and word forms are found in very small areas (between villages less than 40 km apart). The base dialects hardly changed since the last century in this area^48^, necessitating switching abilities of the dialect-competent speakers in order to be understood.

Non-dialect speakers (N = 23) were selected by an open call at the University of Marburg (Germany). These speakers were selected on the basis that they did not speak or comprehend a local Hessian (base) dialect. Both groups were matched regarding age, gender, and education (see Supporting Appendix, Table S1). Dialect competence was assessed by the diphthong test developed by Schmidt and colleagues^44,45^, and dialect dominance (bias) was approximated by an adapted version of the Bilingual Language Profile (aBLP)^16,49^. Both groups underwent a battery of cognitive tests. High-resolution structural brain images were recorded with MRI for GMV and CT analyses. We expected that brain morphology of competent dialect speakers differs from brain morphology of the matched controls in brain regions associated with phonological expertise and language control in bilinguals (e.g., auditory and frontal cortex). Furthermore, we hypothesized that—provided the structural differences exist—brain morphology in the identified regions covaries with dialect competence as measured by the diphthong test. We would therefore contribute to consolidate the view that the acquisition of a dialect resembles the acquisition of a language regarding the repercussions for brain structure. Dialect competence is then an aspect of multilingualism as envisaged by Berthele^12^ and modelled by Pliatsikas^46^.

## Results

### Dialect tests

**Fig. 1:**
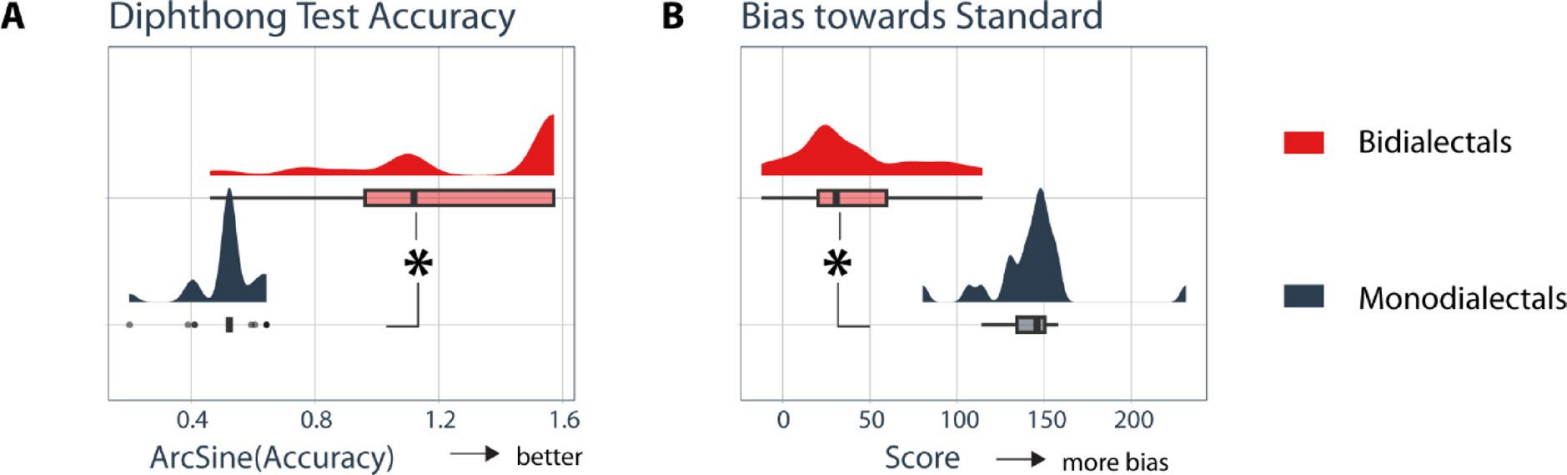
Overview of language test performance of bidialectals (red) and monodialectals (black). (**A**) Raincloud distribution plot for the diphthong test accuracy. (**B**) Raincloud distribution plot for the bias towards Standard German. The asterisks indicate significant differences between the arithmetic means.

The Analysis of Covariance (ANCOVA) on the arcsine-transformed diphthong test accuracy showed a main effect of group (F(1,46) = 77.12, *p* < 0.001, η^2^p = 0.63). A post-hoc test revealed that bidialectal speakers showed a significantly better performance of the diphthong test than monodialectal speakers (t(46) = 8.78, *p* < 0.001, d = 2.52, see Fig. 1A). The covariate age was not significant (F(1,46) = 1.41, *p* = 0.24, η^2^p = 0.03).

The ANCOVA on the aBLP score (adjusted bilingual profile^50^, expressed as bias towards Standard [German]) yielded a main effect of group (F(1,46) = 136.76, *p* < 0.001, η^2^p = 0.75), with monodialectals showing a stronger dominance of Standard German (t(46) = 11.7, *p* < 0.001, d = 3.36, see Fig. 1B). The covariate age was not significant (F(1,46) = 1.20, *p* = 0.28, η^2^p = 0.03).

Diphthong test performance was inversely correlated with the aBLP score (r = – 0.75, df = 47, *p* < 0.001).

The analyses showed that bidialectals differed from monodialectals on the basis of their dialect competence and usage. This outcome was expected and confirmed the validity of our participant selection.

### Cognitive test battery

Bidialectals and monodialectals did not differ in the speech-in-noise, Erikson flanker task and forward digit span task. There was, however, a trend for an effect in the accuracy of the Stroop task. Here, bidialectals were more accurate than monodialectals.

### Brain morphology

**Fig. 2:**
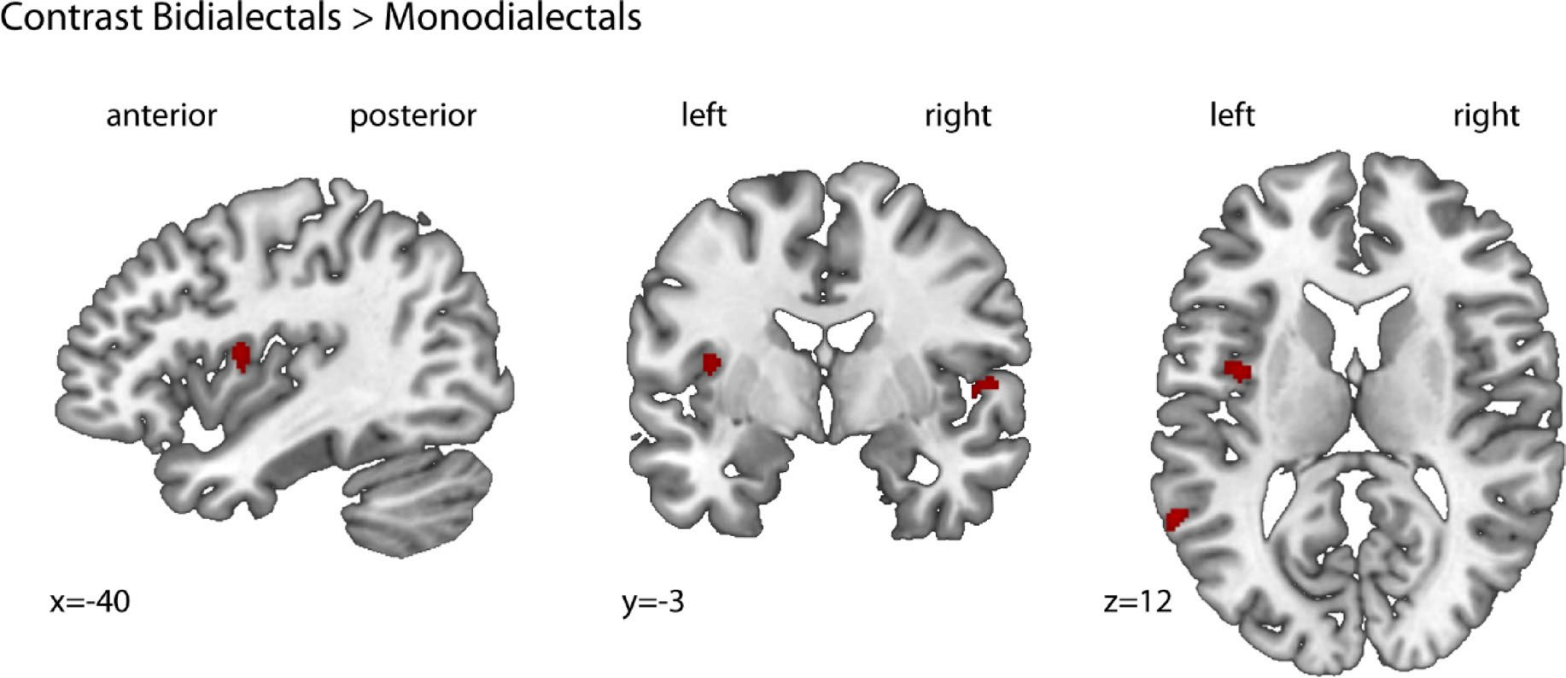
Illustration of significant clusters where gray matter volume is larger in bidialectals than in monodialectals. Coordinates are given in the Montreal Neurological Institute (MNI) space.

In the group whole-brain comparison of volumetric data, several clusters (at *p* < 0.001, extent threshold k = 40) emerged. Gray matter volume (GMV) was larger in bidialectals than in monodialectals in bilateral middle temporal gyrus, extending into superior temporal sulcus, and in bilateral insula, comprising parts of the Rolandic operculum and reaching into Heschl’s gyrus (particularly on the right hemisphere, see Table 1 and Fig. 2).

**Table 1:** Clusters emerging from group comparisons of volumetric and surface data. Both contrasts concern the direction bidialectals > monodialectals. Abbreviations: l.–left, r.–right, MTG–middle temporal gyrus, STS–superior temporal sulcus, Rolandic op–Rolandic operculum, HG–Heschl’s gyrus, SFG–superior frontal gyrus.

*Volume*								
	Cluster (voxels)	Cluster (mm^3^)	Cluster (p uncorr.)	Peak T	Peak MNI (mm)			
	308	462	0.009	6.42	− 60	− 50	8	l. MTG, l. STS
				3.84	− 51	− 42	2	
				3.71	− 66	− 57	− 2	
	134	201	0.065	5.05	70	− 32	2	r. MTG, r. STS
				4.46	70	− 24	− 6	
	81	122	0.142	4.33	54	− 4	3	r. Insula, Rolandic op, HG
	91	137	0.121	4.26	− 39	− 2	12	l. Insula, Rolandic op
*Thickness*								
	Cluster (vertices)							
	34		0.341	3.98	12	20	− 17	r. SFG orbital

No clusters emerged in the opposite GMV contrast (i.e., monodialectals > bidialectals).

**Fig. 3:**
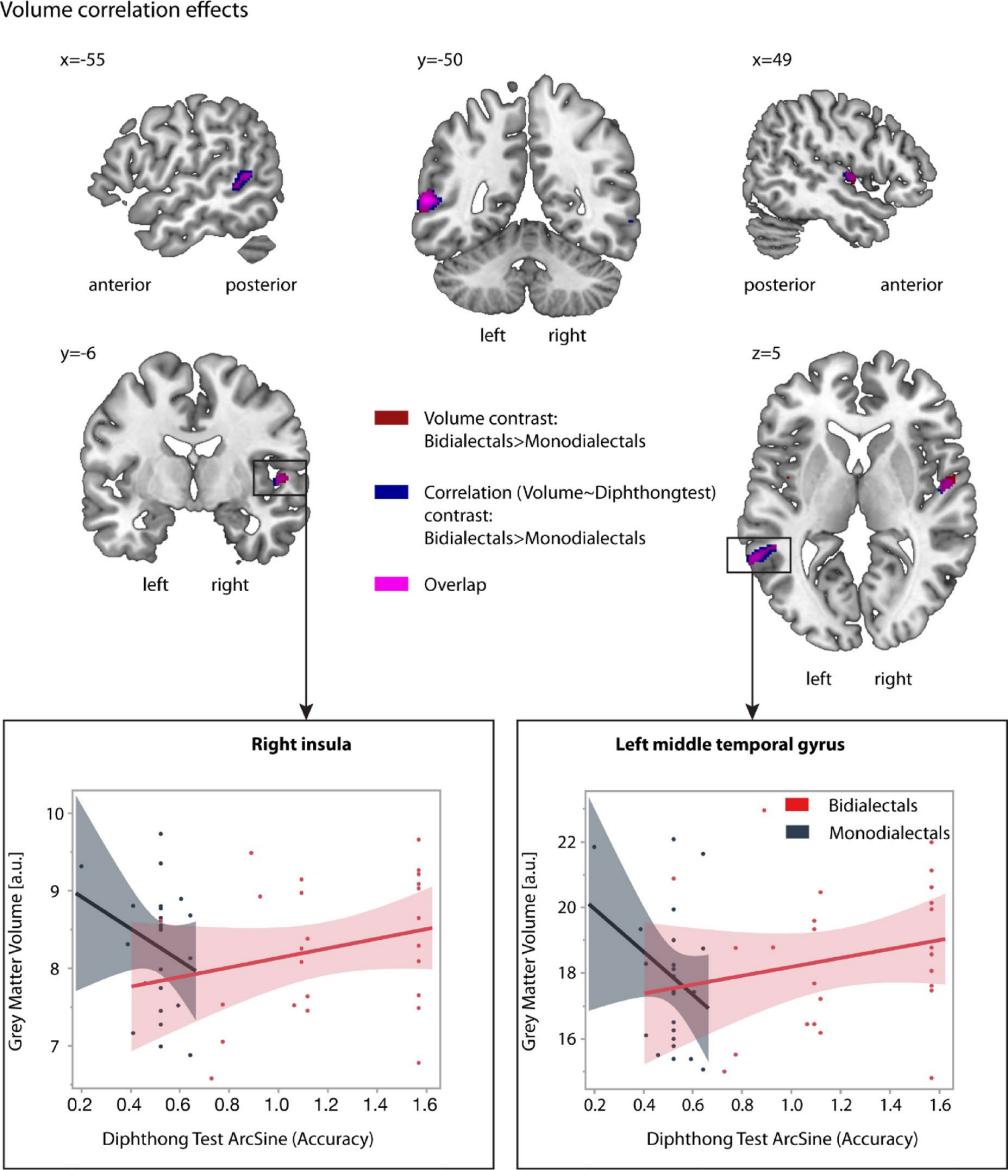
Illustration of clusters where the gray matter ~ diphthong test accuracy correlation was more positive for bidialectals than for monodialectals, overlaid on clusters from the group comparison (bidialectals > monodialectals). Mean GMV values from individual participants parcellated brain areas (right insula, left middle temporal gyrus) are plotted against diphthong test accuracy to illustrate the correlation differences.

The ANCOVA, including no collinear regressors, also allowed us to assess where correlations between regressors of interest and volume or surface differed between bidialectals and monodialectals. Regarding GMV, we found several clusters where the correlation between volume and diphthong test accuracy was more positive for bidialectals than for monodialectals. The largest clusters were found in bilateral middle temporal gyrus and superior temporal sulcus. Two smaller clusters were found in right insula and supplementary motor area, see Fig. 3 and Table 2). There were also clusters where the correlation between volume and speech-in-noise accuracy was more positive for bidialectals than for monodialectals (Table 4). The largest clusters were again in bilateral middle temporal gyrus. A further cluster emerged in left inferior frontal gyrus (comprising pars triangularis and opercularis) and extending into insula (see Table 4). For the remaining regressors, no clusters emerged.

**Table 2:** Clusters emerging from the comparison of the diphthong test accuracy ~ GVM and diphthong test accuracy ~ CT correlations between bidialectals and monodialectals. The cluster reflect areas where the respective correlations with diphthong test accuracy were stronger for bidialectals than for monodialectals. Abbreviations: l.–left, r.–right, MTG–middle temporal gyrus, STS–superior temporal sulcus, SMA–supplementary motor area, Rolandic op–Rolandic operculum, HG–Heschl’s gyrus.

*Volume*									
	Cluster (voxels)	Cluster (mm^3^)	Cluster (p uncorr.)	Peak T	Peak MNI (mm)				
	421	632	0.003	7.27	− 60		− 50	8	l. MTG, l. STS
				4.06	− 54		− 44	3	
	286	429	0.011	4.89	70		− 26	− 8	r. MTG, r. STS
				4.66	70		− 32	2	
				4.35	60		− 46	− 4	
	64	96	0.188	4.35	8		− 12	48	r. SMA
	76	114	0.154	4.21	50		− 6	2	r. Insula Rolandic Op, HG
*Thickness*									
	Cluster (vertices)								
	67		0.12	4.09		35	− 29	− 25	r. Fusiform

**Table 3:** Cluster emerging from the whole brain regression analysis of GMV/CT and diphthong test accuracy on bidialectals only. Abbreviations: l.–left, r.–right, SFG–superior frontal gyrus, ITG–inferior temporal gyrus, SPC–superior parietal cortex, IFG–inferior frontal gyrus, trian–pars triangularis, operc–pars opercularis, MFG–middle frontal gyrus.

*Volume*								
	Cluster (voxels)	Cluster (mm^3^)	Cluster (p uncorr.)	Peak T	Peak MNI (mm)			
	51	77	0.195	5.45	− 24	− 2	− 42	l. Fusiform
*Thickness*								
	Cluster (vertices)							
	68		0.069	6.37	32	22	4	r. Insula
	115		0.011	6.37	− 9	42	23	l. SFG
	43		0.185	5.69	21	33	43	r. SFG
	78		0.047	5.33	48	− 61	− 7	r. ITG
	32		0.285	4.98	18	− 48	67	r. SPC
	44		0.178	4.9	40	− 64	− 18	r. Fusiform
	126		0.007	4.88	− 37	13	25	l. IFG (trian)
				4.15	− 40	2	28	l. IFG (operc)
	71		0.062	4.69	− 57	− 59	3	l. ITG
	78		0.047	4.59	− 30	21	7	l. Insula
	32		0.285	4.04	36	21	50	r. MFG

**Table 4:** Cluster emerging from the whole brain regression analysis of GMV with speech-in-noise accuracy. The cluster reflect areas where the respective correlations with speech-in-noise accuracy were stronger for bidialectals than for monodialectals. Abbreviations: l.–left, r.–right, MTG–middle temporal gyrus, STS–superior temporal sulcus, IFG–inferior frontal gyrus, trian–pars triangularis, operc–pars opercularis.

*Volume*								
	Cluster (voxels)	Cluster (mm^3^)	Cluster (p uncorr.)	Peak T	Peak MNI (mm)			
	511	767	0.001	6.55	− 60	− 48	8	l. MTG, l. STS
				3.45	− 68	− 56	2	
	776	1164	0	5.09	57	− 42	4	r. MTG, r. STS
				4.8	50	− 44	8	
				4.73	52	− 42	20	
	247	371	0.016	4.34	− 45	20	0	l. IFG (triang)
				4.14	− 33	21	4	l. Insula
				3.91	− 50	14	3	l. IFG (operc)

**Fig. 4:**
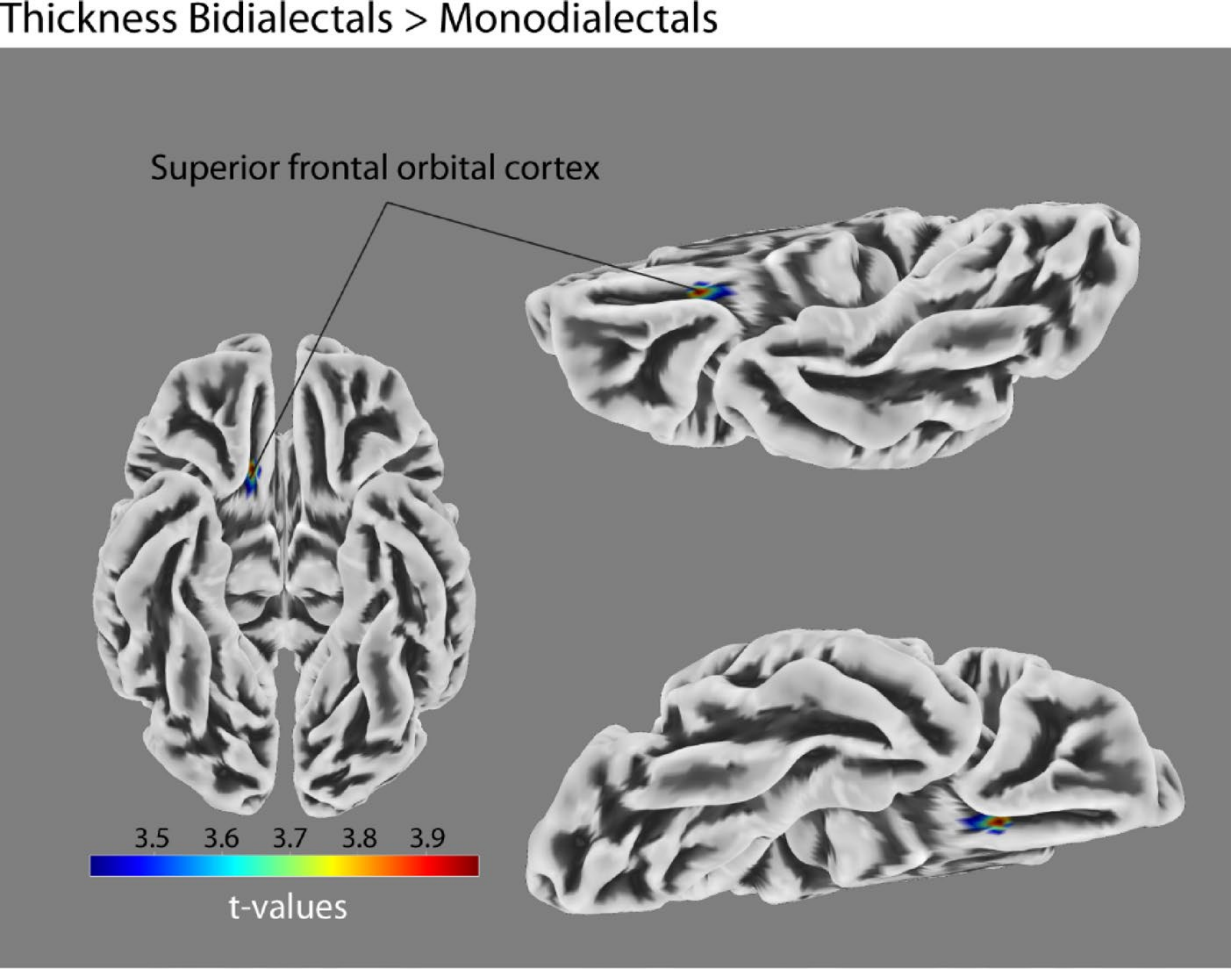
Illustration of cortical surface (thickness) differences in the bidialectals > monodialectals contrast.

For cortical thickness, one cluster emerged where bidialectals showed greater thickness than monodialectals. This cluster was in right orbitofrontal cortex. Again, there were no significant clusters in the opposite thickness contrast (i.e., monodialectals > bidialectals, see Fig. 4 and Table 1 2).

**Fig. 5:**
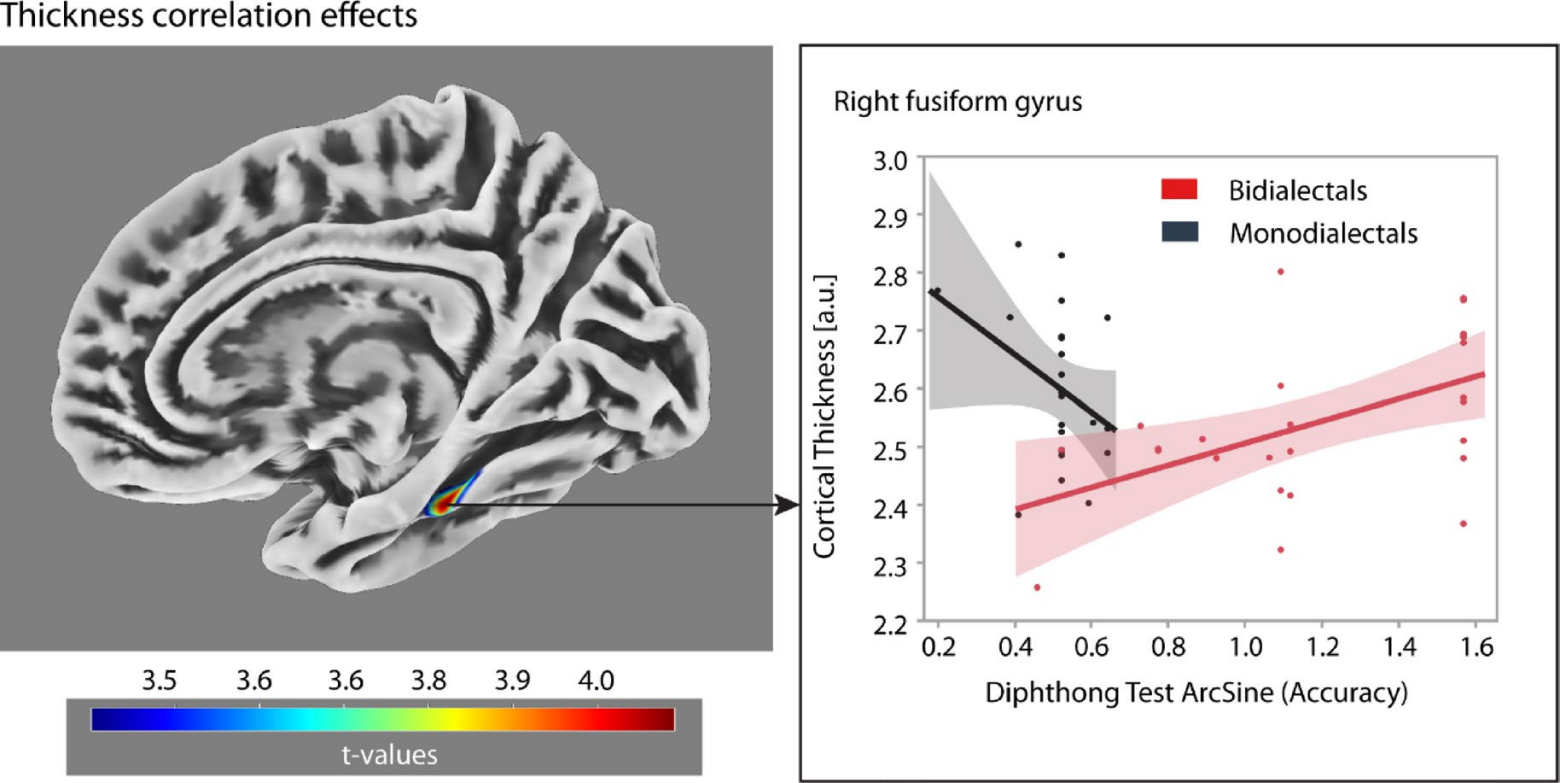
Illustration of clusters where the thickness ~ diphthong test accuracy correlation was more positive for bidialectals than for monodialectals. Mean CT values from individual participants’ parcellated brain areas (right fusiform gyrus) are plotted against diphthong test accuracy to illustrate the correlation differences.

Correlations of CT with diphthong test accuracy were more positive for bidialectals than for monodialectals in right fusiform gyrus (see Fig. 5 and Table 2). There were no further clusters resulting from correlations with the remaining regressors.

**Fig. 6:**
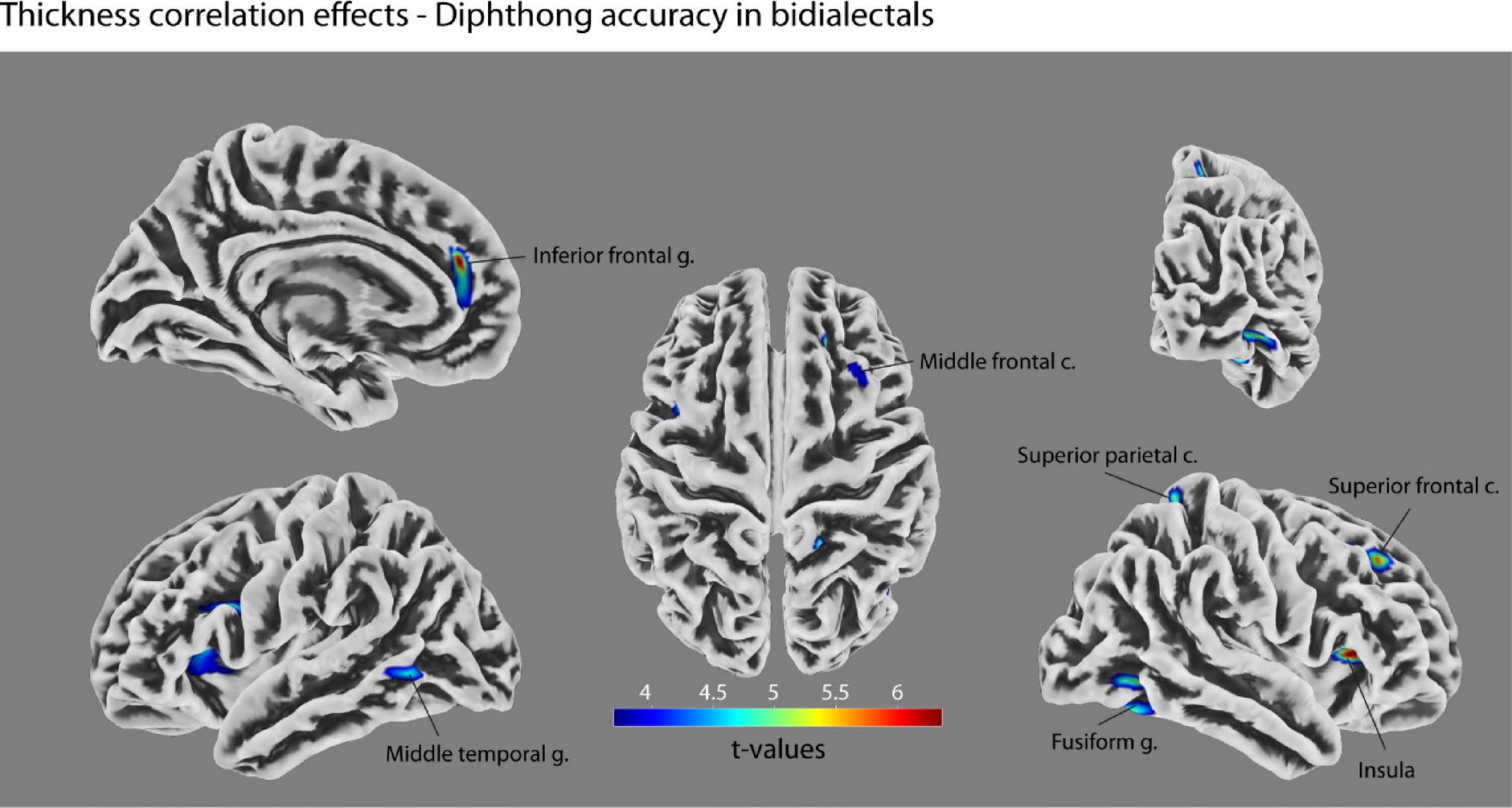
Illustration of clusters where CT correlated with accuracy in the diphthong test for bidialectal participants.

Finally, we ran additional whole-brain multiple regression analyses on bidialectals only. Correlations between diphthong test accuracy and volume revealed a cluster in left fusiform gyrus (see Fig. 6 and Table 3). Correlations between diphthong test accuracy and thickness revealed clusters in bilateral insula, bilateral superior frontal gyrus, bilateral inferior temporal gyrus, right superior parietal cortex, right fusiform gyrus, right middle frontal gyrus and left inferior frontal gyrus (see Fig. 6 and Table 3). Correlations between speech-in-noise accuracy and volume showed clusters in left inferior frontal gyrus, right precuneus and bilateral middle temporal gyrus. Correlations between speech-in-noise accuracy and thickness resulted in clusters in bilateral insula, right Heschl’s gyrus and right inferior parietal cortex (see Table 5).

**Table 5:** Cluster emerging from the whole brain regression analysis of GMV/CT and speech-in-noise accuracy on bidialectals only. Abbreviations: l.–left, r.–right, IFG–inferior frontal gyrus, operc–pars opercularis, MTG–middle temporal gyrus, STS–superior temporal sulcus, HG–Heschl’s gyrus, IPC–inferior parietal cortex.

*Volume*								
	Cluster (voxels)	Cluster (mm3)	Cluster (p uncorr.)	Peak T	Peak MNI (mm)			
	67	101	0.141	6.67	− 51	12	3	l. IFG (operc)
	64	96	0.15	5.45	8	− 52	68	r. Precuneus
	61	92	0.159	5.45	− 62	− 46	9	l. MTG, l. STS
	51	77	0.195	4.69	54	− 42	18	r. MTG, r. STS
*Thickness*								
	Cluster (vertices)							
	127		0.007	9.98	32	22	4	r. Insula
	68		0.069	5.05	− 30	21	7	l. Insula
				3.82	− 35	9	12	
	39		0.216	4.33	34	− 28	14	r. HG
	33		0.274	4.3	48	− 49	41	r. IPG

In sum, bidialectals differed from monodialectals in brain morphology, both in GMV and CT, in frontal and temporal brain regions associated with language control in bilinguals. Furthermore, the correlation analyses showed that structural differences in these areas co-varied with dialect competence as measured by the diphthong test. Participants also underwent a battery of cognitive tests (see Supporting Appendix). Bidialectals did not significantly outperform monodialectals in these measures, though.

## Discussion

Bidialectals with an attested dialect competence and a bias towards their local dialects (rather than Standard German) differed from monodialectals without dialect competence and a bias towards Standard German on the basis of their brain morphology. The structural differences concerned both gray matter volume (GMV) and cortical thickness (CT), which was larger in bidialectals than monodialectals in temporal and frontal regions. Notably, the temporal regions showed a stronger correlation of GMV with accuracy in the diphthong test. A viable conclusion is therefore that the attested difference in brain morphology is based on experience and not due to a random effect of selecting participants with comparable brain structure. Dialect competence therefore seems to have repercussions for brain structure and brain plasticity.

The largest clusters found in our study comprised (posterior) middle temporal gyrus bilaterally, extending (on the right hemisphere) into superior temporal sulcus (STS). The posterior STS has been identified as part of the phonological network^51–53^, while the posterior part of the middle temporal gyrus has been associated with semantic processing^54^. It is reasonable to assume that dialect competence has repercussions for the phonological system and the sound-meaning mapping. Alternative word forms of one concept (as required to be accessed during the diphthong test) are likely to be represented in these areas. Therefore, both larger GMV as well as stronger correlations of GMV with diphthong test accuracy provide brain structural evidence for larger phonologies of bidialectals. Regarding bilingualism, the middle temporal gyrus (bilaterally) seems to be similarly involved in phonological and lexico-semantic tasks, with differing activations in L1 and L2^24^, but also in general lexico-semantic processing^55^.

The insula has been identified as a core region of the language processing network^56^ and is part of the dorsal stream^57^, associated with the mapping of articulatory to sensory information. To this end, evidence has been provided that the insula supports articulatory planning^58^ and articulatory control^59^. Importantly, the insula is also a key region of the bilingual network^24^. In the meta-analysis of Sulpizio, et al.^24^, the insula emerged either in the L1 > L2 (first language > second language) contrast when focusing on grammar: i.e., was more strongly involved in L1 than in L2 grammar processing. The opposite contrast (L2 > L1) also emerged when focusing on lexico-semantic processing: i.e., was more strongly involved in L2 than in L1 lexico-semantic processing. Crucially, the insula plays an important role in language switching^24,60^ and language conflict resolution^61^.

There are much less studies within bilingualism research which focus on structural properties of the insula. To this end, Rodriguez, et al.^33^ showed that left insula structure predicts speech sound learning abilities in bilinguals, but not in monolinguals. However, in this study, bilinguals with good sound learning abilities showed cortical thinning, compared to those with worse sound learning abilities, i.e., the correlation between sound learning and cortical thickness was negative. On the other hand, Li, et al.^62^ reported increased gray matter volume in insular cortex for bilinguals compared to monolinguals and Golestani and Pallier^63^ found a positive correlation of gray matter volume in left insula and quality of foreign speech sound production.

In the current study, we not only found increased volume of the insula in bidialectals compared to monodialectals, but also positive correlations for both volumetric and thickness measures with dialect competence as measured by the diphthong test. The diphthong test, on which basis bidialectals and monodialectals differed, essentially tests the appropriate choice of word forms, or more precisely, the correct selection of a diphthong (such as [a͡ɪ]) or a long vowel (such as [iː]) in potentially conflicting forms between dialect and Standard German. Therefore, the test requires contextually appropriate response selection and conflict resolution, abilities which are supported by the insular cortex^64–66^. Bidialectals are confronted more often with a contextually appropriate response selection and conflict resolution due to their dialect usage experience. It is thus reasonable to assume that this skill affects the cortical structure in insular cortex. Our interpretation is further corroborated by studies which suggest that the insular cortex is also involved in processing speech sounds with enhanced phonological complexity^67^, such as diphthongs, and that insular cortex is sensitive to the place of articulation of speech sounds^68^, which would differ between the diphthong and the long vowel.

Bidialectals differed from monodialectals in thickness of the orbitofrontal context (superior frontal gyrus). A similar cluster emerged from the thickness-diphthong test accuracy correlation, again suggesting that the group difference in brain structure was not random, but is associated with a specific language (dialect) skill. The aforementioned clusters coincide with the right dorsolateral prefrontal cortex^69^, an area that has been attributed both domain general and domain specific (i.e., language-based) function. Regarding domain general functions, the area has been identified to support working memory, executive control and response inhibition^70–72^. With respect to language functions, the region was attributed several functions supporting bilingual language processing. Summarized in Sulpizio, et al.^24^, both superior and middle frontal cortex are involved in language switching and more engaged in L2 phonological processing. As with left insula, we also found that superior and middle frontal cortex showed significant correlations between gray matter volume (and, to a lesser degree, cortical thickness) and diphthong test accuracy. It seems that the functional interpretation is similar to that of left insula: Since these areas of the prefrontal cortex are engaged in supporting flexible and adaptive behavior, with adaptions to changes in communication and response inhibition^70,71^, they thereby support the abilities necessary for the diphthong task in which participants have to select the appropriate word form, dependent on the context (here: dialect).

The remaining clusters where GMV or CT correlated with diphthong test accuracy are all parts of the bilingual network as illustrated by Sulpizio, et al.^24^. The inferior parietal cortex and the fusiform gyrus have been found to be implicated in language switching in bilinguals, but also in lexico-semantic and phonological tasks, with either stronger activity in L1 or L2^24^. The inferior frontal gyrus has similarly been found to support lexico-semantic and phonological processing to different degrees in bilinguals as compared to monolinguals^24^. The GMV cluster in right Heschl’s gyrus, where bidialectals differed from monodialectals and showed a stronger correlation with the diphthong test accuracy, corroborates previous studies on bilingualism and multilinguals which found structural differences in auditory cortex^36,37^. Again, this can be interpreted with respect to the specifics of the diphthong task: Selecting the appropriate speech sound necessitates accurate representation and processing. Thus, larger cortical structure in auditory cortex seems to support this skill.

Another skill that covaried differently with brain structure in bidialectals and monodialectals was speech-in-noise accuracy. Bidialectals, compared to monodialectals, showed stronger correlations with CT in temporal, frontal and parietal regions, all part of the bilingual language network^24^. Since some of the clusters overlapped with clusters where bidialectals differed from monodialectals (e.g., insula, superior frontal gyrus), we suggest that bidialectals have at their disposal enhanced cortical structures to support speech processing in challenging contexts. Note that a general difference in speech-in-noise accuracy was not found, most likely due to the fact that both groups were tested on Standard German. This issue should be resolved in future research.

Altogether, the pattern of GMV and CT findings suggests that bidialectals differ from monodialectals in in ways similar to how bilinguals differ from monolinguals when considering brain structure, complementing and extending previous findings^42^. Parts of the bilingual network therefore show comparable plasticity with respect to dialect experience and competence. These findings are compatible with the »Dynamic Restructuring Model«^46^. As measured by the diphthong test, dialect competence is experience in selecting appropriate word forms with the respective correct speech sounds. This experience results in brain plasticity. On this background, the acquisition of a dialect has similar repercussions for brain structure than the acquisition of a language.

## Conclusion

The ability to be competent in a dialect, next to being fluent in a (standard) language, has repercussions for the cortical language network. We here provided evidence that bidialectals, compared to monodialectals, showed larger GMV and CT in regions associated with the bilingual language network. In particular, these regions support appropriate language selection (switching) and task-specific control in mapping between auditory and articulatory information on the one hand and between auditory and semantic information on the other hand. In the light of negative attitudes against certain dialects^73^, this study provides a counter-weight in providing evidence that competence in any dialect can result in experience-dependent brain structural adaptations, allowing for flexible context-appropriate communicative behavior.

## Materials and methods

### Participants

#### Dialect speakers (Bidialectals)

We recruited 26 dialect speakers (14 females, 12 males) from Northwestern Hesse, with support by contact persons in the local dialect association (https://dialektverein.de/). Participants stemmed from areas of central and northern Hesse, as well as the transition zone between the two, according to Wiesinger^74^. Inclusion criteria were that participants were older than 18 and younger than 60, had lived at least 20 years in the Northwestern Hesse area and were dialect competent according to their self-disclosure. Bidialectals were on average 36 years (SD = 11 years, minimum = 18 years, maximum = 58 years).

#### Non-dialect speakers (Monodialectals)

Another 23 non-dialect speakers (13 females, 10 males) were recruited from the area around Marburg and Gießen. The same inclusion criteria as for the bidialectals applied, except that it was required that they did not report dialect competence in their self-disclosure. Furthermore, it was also required that participants’ parents did not speak a local dialect. Monodialectals were on average 34 years (SD = 12 years, minimum = 19 years, maximum = 54 years). More detailed demographic information is provided in Table S1.

#### Ethics statements (all participants)

All participants gave their informed written consent prior to their participation in any part of this study. Participants were informed about all details of the study and the safety protocols applying to the recording in the MR scanner. The study was in accordance to the declarations of Helsinki approved by the Ethics Committee of the Linguistic Society of Germany (DGfS, votum #2019-08-190912).

#### Comparison of participant groups (demographics)

Participant groups did not differ in their proportions of females and males (χ^2^ = 0.04, df = 1, *p* = 0.85), or in their age (t = 0.61, df = 47 *p* = 0.54). They also did not differ regarding the years they learned their second language (English, t = 0.54, df = 47, *p* = 0.59) or in the proportion of having learned a third language (χ^2^ = 2.11, df = 1, *p* = 0.15). Finally, they did not differ on the basis of years of education (t = 1.50, df = 47, *p* = 0.14).

Participant groups did differ, however, regarding the age of onset of Standard German (t = 2.37, df = 47, *p* < 0.05), with monodialectals yielding an earlier onset than bidialectals. Furthermore, the groups also differed in their dialect biases, as assessed by the aBLP (t = 11.81, df = 47, *p* < 0.001). The monodialectals showed a much stronger bias towards Standard German.

#### Significance statement

We here show that persons being competent in a local dialect, alongside being fluent in their standard language, differ from non-dialect speakers in ways similar to how bilinguals differ from monolinguals. These differences were seen as brain structural differences in the bilingual network, particularly in frontal networks engaged in code switching. Dialect competence is thus based on brain plasticity, comparable to findings from bilingualism research.

### Language tests

Language skills and dialect competence were assessed by two surveys, the adjusted Bilingual Language Profile (aBLP) and the diphthong test.

#### Adjusted bilingual language profile (aBLP)

The Bilingual Language Profile (BLP) is an instrument for assessing language dominance (bias) in bilinguals^50^. The BLP is available in open-access format and consists of 19 self-report items that relate to language history, language use, language proficiency and language attitude. The BLP has previously been used to assess bias in bidialectals^16^. Following these authors, we used the adjusted BLP (aBLP) for calculating the dominance of either Standard German or the respective dialect of the participants of our study.

Participants filled out the 19 items in a paper-and-pencil version of the aBLP. Responses were scored according to the tutorial provided by Birdsong and Gertken^50^. By-participants scores were then used for further analyses.

#### Diphthong test

The »diphthong test« is a dialect competence measure developed at the research center »Deutscher Sprachatlas«^44,45^. The test assesses the ability of participants to produce the correct phonetic form of words in the respective local dialect. Knowledge about the expected target forms goes back to an exhaustive dialect survey by Georg Wenker in the late nineteenth century. This knowledge was corroborated by subsequent surveys and audio recordings in the twentieth and twenty-first century within the project “Regionalsprache.de”^43^.

For the purpose of the diphthong test, target nouns of the survey material were selected which contained the diphthong [a͡ɪ], as in modern English *ice* or Standard German *Eis.* A total of 10 nouns were selected. In half of these nouns, the diphthong relates to a diphthong in Middle High German (MHG). In the other half of these nouns, the diphthong relates to the long vowel [iː] in MHG (see Table S2 for details). All nouns were displayed on a computer screen and participants had to pronounce them in their local dialect. Pronunciations were recorded by a Sennheiser EM 9600 microphone and digitized at 44.1 kHz sampling rate and 16 bit amplitude resolution. Recordings were stored as WAV-files on a personal computer, running Windows 7. Recordings were subsequently analyzed manually. Target words (see Table S2) were compared to the expected target pronunciation of the local dialect of participants. Local dialect was determined as dialect of the place a participant spend most time within the first 20 years of life. For each participant, a competence score was calculated. To this end, if all target words were correct, the score was 100% (10 out of 10). Incorrect target words could deviate from the expected local dialect pronunciation in either the onset or offset of the diphthong, or included an erroneous monophthong (single vowel). The number of correct words was then divided by the total number of produced target words and multiplied by 100. If all target words were produced, this number was 10, if specific target words were not produced, this number decreased accordingly. Percentages of correct pronunciations were then arc-sine transformed for further analyses^75^.

#### Extended cognitive test battery

To follow a standard practice in bilingual research, we added an extended cognitive test battery (details are reported in the Supplementary Information). The test battery included a speech-in-noise task, the Erikson Flanker task, the Stroop task and the forward digit span task.

### Structural scans

#### Recording

Brain structural images were acquired on a Siemens Trio (A Tim System, 3 Tesla) with software version Syngo MR B17. Images were T1-weighted anatomical scans acquired with a 32-channel head coil using an MPRAGE sequence. The acquisition parameters of the images were as follows: 3D sagittal acquisition, 176 slices with 1 mm thickness, field of view of 256 × 256 mm, matrix of 246 × 256, resulting in isotropic voxels of 1 × 1 × 1 mm^3^, TR = 1900 ms, TE = 2.52 ms, TI = 900 ms, flip angle = 9, pixel bandwidth = 170 Hz.

#### Preprocessing

Raw pictures in the DICOM format were preprocessed using the pipeline of the Computational Anatomical Toolbox (CAT12, Gaser, et al.^76^) for SPM12 (Welcome Trust Center for Neuroimaging, cf. Ashburner^77^), implemented within Matlab R2024A (Mathworks, Nattick, MA, USA). We followed the default procedure suggested in the manual (https://neuro-jena.github.io/cat12-help/) for both volumetric (VBM) and surface-based (SBM) morphometry.

For VBM, the preprocessing steps involved tissue class segmentation, registration to a standard template (in MNI space), spatial normalization of the gray matter maps, and interpolation to a standard voxel size of 1.5 × 1.5 × 1.5 mm^3^. We selected the Thalamic Nuclei Atlas^78^, the Neuromorphometrics Atlas (included in SPM) and the Automated Anatomical Labelling Atlas (AAL3, Tzourio-Mazoyer, et al.^79^). A final data check confirmed that for all participants, quality was acceptable, with Brain Web Phantom biases below 80% or a nominal quality rating of at least B- ^80^. Since the standard pipeline does not involve a correction for head size or volume, we extracted the total intracranial volume for all participants and corrected subsequent volumetric statistical analyses by removing any variance that can be explained by TIV. The final step of VBM preprocessing involved smoothing with a 6 mm full-width at half-maximum (FWHM) Gaussian kernel.

For SBM, surface parameter were extracted from anatomical scans, using the standard settings of the preprocessing pipeline (https://neuro-jena.github.io/cat12-help/). Surface data were smoothed with a 15 mm full-width at half-maximum (FWHM) Gaussian kernel. Subsequently, these data were used for estimating cortical thickness.

### Statistical analyses

Statistical analyses of language performance and cognitive test data were carried out in the open access statistical software jamovi (The jamovi project, 2025. jamovi (Version 2.6) [Computer Software]. Retrieved from https://www.jamovi.org). For reasons of comparison, group comparisons were based on ANCOVAs, where participant age was always included as covariate (similar to the analyses of volumetric and surface data). Effect sizes for main effects are given as partial eta-squared (η^2^p), effect sizes for planned comparisons as Cohen’s d.

Statistical analyses of pre-processed volumetric and surface data were carried out in CAT12. We first analyzed group-differences in volume between mono- and bidialectals. To this end, a full factorial model (corresponding to an ANCOVA) was set up, with group as factor, including the following covariates: age, gender, digit span score, Flanker effect (accuracy), Flanker effect (reaction time), Stroop effect (accuracy), Stroop effect (reaction time), speech-in-noise accuracy, diphthong test accuracy, bias towards Standard. As this model yielded a strong collinearity effect between diphthong test accuracy and bias towards Standard, the variable bias was removed from the final model. TIV was corrected by the ANCOVA-option in the standard CAT12 pipeline (https://neuro-jena.github.io/cat12-help/). Furthermore, an implicit masking with the absolute threshold of 0.2 was used. The value represents the fractional tissue probability and lies within the recommended range of 0.1–0.2. Contrasts were defined in order to assess volumetric differences on a whole-brain level between groups, and correlational differences with covariates between groups (i.e., interactions). Contrasts were calculated at *p* < 0.001 (uncorrected) and an empirically determined cluster extent threshold. A reduced model (identical to the aforementioned model but without the group factor, i.e., essentially, a multiple regression) was set up for bidialectals only (i.e., without interactions). Anatomical locations were identified on the basis of MNI peak coordinates per cluster, looked up in the AAL3 atlas and checked against locations determined from the Neuromorphometrics Atlas. For illustration, mean gray-matter volume was extracted from the anatomical locations identified by clusters from the whole-brain analyses on the basis of the AAL3 atlas.

In a similar way, surface-based data for estimating CT were analyzed. Again, a full-factorial model (ANCOVA) was set up, with the same factors and covariates as for the volumetric model. Crucially, however, there was no TIV correction and implicit masking without absolute threshold. Specifications of the surface model were based on the CAT12 standard pipeline recommendations (https://neuro-jena.github.io/cat12-help/). Specific contrasts were calculated at *p* < 0.001 (uncorrected). As with the volumetric model, a reduced surface model (a multiple regression) was set up for bidialectals only. Anatomical locations were identified on the basis of MNI peak coordinates per cluster, looked up in AAL3 atlas and checked against the information from the Destrieux Atlas ^81^. For illustration, mean thickness was extracted from the anatomical locations identified by clusters from the whole-brain analyses on the basis of the Destrieux Atlas.

## Supplementary Information

Below is the link to the electronic supplementary material.

Supplementary Material 1

## Data Availability

The datasets generated during and analyzed during the current study are available in the following osf-archive: (10.17605/OSF.IO/64D9W) Further analyzes, tables and figures are available in the Supplementary Information (SI).
