# Supplementary material for: Brain-structural differences underlying dialect competence in the bilingual network
Source: Sci Rep. 2026 Jul 1;16:20055. doi: 10.1038/s41598-026-59884-y (PMC13324526; doi:10.1038/s41598-026-59884-y)
Supplement: Supplementary file 1 — Supplementary Material 1 [file 41598_2026_59884_MOESM1_ESM.docx]

**Supplementary Information**

Brain-structural differences underlying dialect competence in the bilingual network

Mathias Scharinger^1,2,3^, Jürgen E. Schmidt^2^, Jens Sommer^3,4^, Andreas Jansen^3,4^

^1^Research Group »Phonetics«, Institute of German Linguistics, Marburg University, Germany

^2^Research Center »Deutscher Sprachatlas«, Marburg University, Germany

^3^Center for Mind, Brain & Behavior (CMBB), Universities of Gießen & Marburg

^4^Core-Facility Brain Imaging, Faculty of Medicine, Marburg University, Germany

**Corresponding author:**

*Mathias Scharinger

Marburg University – Pilgrimstein 16 – 35037 Marburg – Germany

Email: mathias.scharinger@uni-marburg.de

Materials and Methods

Extended demographic information

For extended demographic information, participants filled out an additional questionnaire. Therein, they were asked to report whether they spoke a third language (additionally to German as a native language and English as the first foreign language). Furthermore, they were required to indicated how long they had been speaking English. This information is summarized in Table S1.

Extended information about the diphthong test

The diphthong test was based on 10 sentences that should be spoken in the home dialect of participants (dialect group) or in their standard language at home (control group). The key words of these sentences together with the Middle High German origin of the vowels (either diphthongs such as <ei> or monophthongs such as long <i> are given in Table S2.

Cognitive tests

Participants underwent a battery of cognitive tests. This was done in order to enable comparisons to previous work which showed heterogeneous results regarding cognitive skills co-varying with language- or dialect competence ^[1-4]^.

*Speech-in-noise*

The recognition of speech in noise taxes auditory working memory ^[5,6]^ and requires selective attention to the relevant acoustic stream ^[7]^. Speech-in-noise paradigms either focusing on energetic (low-level) or informational (high-level) masking provided some evidence that bilinguals show worse performance in these tasks ^[8]^; however, the so-called “bilingual disadvantage” is alleviated by language proficiency. Altogether, the speech-in-noise paradigm based on energetic masking is a good proxy to assessing auditory working memory performance.

We here used 44 sentences from the German Speech-in-Noise corpus (GSPIN, Erb, et al. ^[9]^), translated from the English corpus provided by Kalikow, et al. ^[10]^. All 44 sentences ended in a low-predictability noun and consisted of a total of 6 words. Sentences were recorded from a phonetically trained female speaker and digitized at 44.1 kHz with an amplitude resolution of 16 bit. Subsequently, uncorrelated (white) noise was generated within MATLAB (v. 2019a, The Mathwork, Inc. Nattick, MA, USA), with the same digitization settings as the speech recordings. The duration of the white noise was 1 sec longer than the duration of the respective sentences. Sentences were embedded in the middle of the white noise recordings such that the white noise preceded and succeeded the speech material by 500 ms. Noise-embedded sentences were multiplied with the first half period of a (1-cos(x))/2 function for fading-in, and with the first half period of a (1+cos(x))/2 function for fading-out. Fade-in and fade-out windows had durations of 100 ms each. The level of the white noise was +5dB above the average level of the speech recordings.

Participants were presented with individual sentences played over earphones (Sennheiser HD 550), controlled by the experimental software OpenSesame ^[11]^. Their task was to orthographically transcribe as accurately as possible the sentence they just heard. The task was self-paced, participants could proceed by clicking on the space button. The intensity level of the audio was adjusted to a comfortable listening level corresponding to ~60 dB sound intensity. From the 44 sentences, 14 sentences were played as practice items, while 30 sentences were played as test items. Each participant received the same practice and test items, but their order was randomized separately for each participant.

Subsequently, participants’ recognition was assessed by hand, using the following scoring system: Correctly identified words were assigned 2 points. Words that slightly differed from the target words were given 1 point. Deviations were tolerated if the identified words had different inflectional endings (e.g., plural instead of singular, or past tense instead of present tense). Incorrectly identified or missed words were assigned 0 points. Since all sentences contained 6 words, the highest score per sentence was always 12. The individual scores were expressed as ratios with respect to the latter number and multiplied by 100, yielding the measure “percentage correct”. For further analyses, percentage correct values were arcsine-transformed ^[12]^.

*Erikson Flanker task*

The Erikson Flanker task is a task assessing inhibitory control (response inhibition) by presenting congruent of incongruent visual letter strings ^[13]^. The task taxes attentional control ^[14]^ and is often used in cognitive test batteries on bilingual participants ^[15]^. The task was recently applied to bidialectal participants ^[3]^ and is thus a feasible way to assess cognitive performance in several participant groups.

We used a simplified version of the task where participants had to correctly identify a target letter flanked by congruent or incongruent letter. We therefore presented 5-letter strings (consisting of “S” or “H” letters, respectively). The middle letter (S or H) could be flanked by either congruent letters on the left or right (i.e., S or H), or by incongruent letters (i.e., H or S).

Letter-strings were presented to participants in the middle of a computer screen in 4 blocks. Each block contained 20 congruent and 20 incongruent trials. Presentation of items was controlled with OpenSesame ^[11]^. Prior to a letter-string, a fixation sign (white dot) was presented for a randomly selected presentation time uniformly drawn from the range between 500 and 1000 ms. The letter string was then presented until participants pressed the required response button, corresponding to the letter in the middle (S or H). After participants’ responses, a blank screen (duration: 500 ms) preceded the next fixation sign. After each block, participants could pause the experiment and resume by a button press. Prior to the test blocks, participants could administer a practice block consisting of 8 items.

Button presses to the test items were subsequently translated into percentages of correct responses and then arcsine-transformed ^[12]^. The Flanker accuracy effect (effect score) was calculated as difference between the arcsine transformed accuracy to the incongruent and the arcsine transformed accuracy to the congruent trials. More positive going effect scores thus indicate less interference effects between the target and the flanking letters. Furthermore, reaction times from correct trials were log-transformed (natural logarithm). Again, a Flanker reaction time (effect score) was calculated by subtracting the log-transformed reaction times of congruent trials from the log-transformed reaction times of the incongruent trials. Here, more positive-going effect scores indicate more interference.

*Stroop task*

The Stroop task (or Stroop color word test) taxes response inhibition and selective attention. By presenting color adjective in either congruent or incongruent color, response conflicts occur if the task is to name the font color of the color adjective and the font color does not match the color denoted by the adjective ^[16,17]^. Together with the Flanker test, the Stroop task has been used in research on bilinguals for assessing cognitive performance, particularly executive control ^[18-20]^.

We used a relatively simple version of this test in which participants had to indicate the font color of three possible color adjectives (“blau” [blue], “gelb” [yellow] and “rot” [red]) by respective button presses on a computer keyboard. Three buttons were selected and color-coded: “n” for yellow, “b” for blue and “v” for red. Note that keyboard letters could not be seen since they were covered by stickers in the colors elucidate above. In the congruent conditions, the font color corresponded to the color denoted by the adjective. In the incongruent conditions, the font color did not correspond to the color denoted by the adjective. We added a third, neutral, condition, in which the sequence “xxx” was presented in either of the three font colors (red, blue or yellow). Experimental items were distributed over 3 blocks, containing 36 items each (12 congruent, 12 incongruent, 12 neutral). In each block, a fixation dot occurred for 200 ms, followed by 200 ms blank screen before the individual color adjectives were displayed in the middle of the screen until participants’ responses were given. After participants’ responses, the next trial started after 1 sec. After each block, participants could pause and resume the experiment by a button press. Prior to the experimental blocks, 18 practice items were presented to familiarize with the experiment. We used OpenSesame ^[11]^ for controlling the presentation of the stimuli.

Percentage of correct responses (to congruent and incongruent trials) were arcsine-transformed ^[12]^. In order to assess the Stroop effect, arcsine-transformed accuracy of congruent trials was subtracted from arcsine-transformed accuracy of incongruent trials, yielding effect scores. More positive-going effect scores indicate less interference regarding the color denoted by the adjective and the color of the font in which the adjective was displayed. Furthermore, we also calculated a Stroop response time effects (effect scores). Log-transformed responses to correct trials in the congruent condition were subtracted from log-transformed responses to correct trials in the incongruent condition. Here, more positive-going effect scores indicate more interference.

*Forward digit span*

The forward digit span test is a subtest of the Wechsler memory scale ^[21]^ and considered a test to assess working and/or phonological memory capacity ^[22-24]^. Since research in bilingualism is interested in potential cognitive effects of bilingual persons, the digit span is often used for assessing cognitive performance in these populations ^[25]^ We focus on a simple version of the test, the visual forward digit span test.

Participants were presented with increasing sequences of visually presented digits on a computer screen, starting with two digits. Sequences were to be remembered while they were displayed on the screen for 5 secs. Afterwards, participants had to type in the series of digits in the exact same (forward) sequence. Upon completion, the next sequence was displayed. If two consecutive sequences were recalled correctly, the sequence of digits was increased by 1, if two consecutive sequences were recalled incorrectly, the sequence of digits was decreased by 1, otherwise, the length of the sequence did not change. The task ended after 20 trials. The length (in digits) of the longest correctly recalled sequence of digits was then used for further analyses.

Results (cognitive tests)

The ANCOVA on the digit span score did not show a group effect (F(1,46)=0.07, p=0.79, η^2^p=0.002, see Figure S1A), nor a significant covariate of age (F(1,46)=0.02, p=0.88, η^2^p=0.001). A similar picture emerged for the Flanker accuracy effect (group: F(1,46)=1.40, p=0.24, η^2^p=0.03, see Figure S1B; age: F(1,46)=2.32, p=0.13, η^2^p=0.05) and the Flanker reaction time effect (group: F(1,46)=0.58, p=0.45, η^2^p=0.01, see Figure S1C; age: F(1,46)=0.20, p=0.66, η^2^p=0.004).

However, there was a group trend for the Stroop accuracy effect (F(1,46)=3.29, p=0.08, η^2^p=0.07), with bidialectals showing a higher effect score than monodialectals (t(46)=1.81, p<0.05, d=0.52, one-sided, see Figure S1D). The covariate age was not significant (F(1,46)=0.42, p=0.52, η^2^p=0.01).

The ANCOVA on the Stroop reaction time effect did not show a group effect (F(1,46)=0.71, p=0.40, η^2^p=0.02, see Figure S1E) nor a significant age covariate (F(1,46)=0.01, p=0.94, η^2^p=0.001), similar to the ANCOVA on speech-in-noise accuracy (group: F(46)=1.43, p=0.24, η^2^p=0.03; age: F(46)=0.17, p=0.68, η^2^p=0.004, see Figure S1F). A summary of the results from the cognitive tests is provided in Table S3.

Cognitive test scores showed some mutual correlations, as elucidated in the following. First, digit span performance positively correlated with the Flanker reaction time effect (r=0.39, df=47, p<0.01) and with speech-in-noise accuracy (r=0.36, df=47, p<0.05). Next, there were also correlations between measures of language (dialect) performance and cognitive tests. Here, the Stroop accuracy effect was negatively correlated with bias towards the standard (r=–0.35, df=47, p<0.05), replicating the pattern of the group differences. Furthermore, speech-in-noise accuracy negatively correlated with accuracy in the diphthong test (r=–0.34, df=47, p<0.05). All correlations are visualized in Figure S2.

In sum, both groups did not differ in their cognitive skills, except when looking at the Stroop test, where bidialectals outperformed monodialectals.


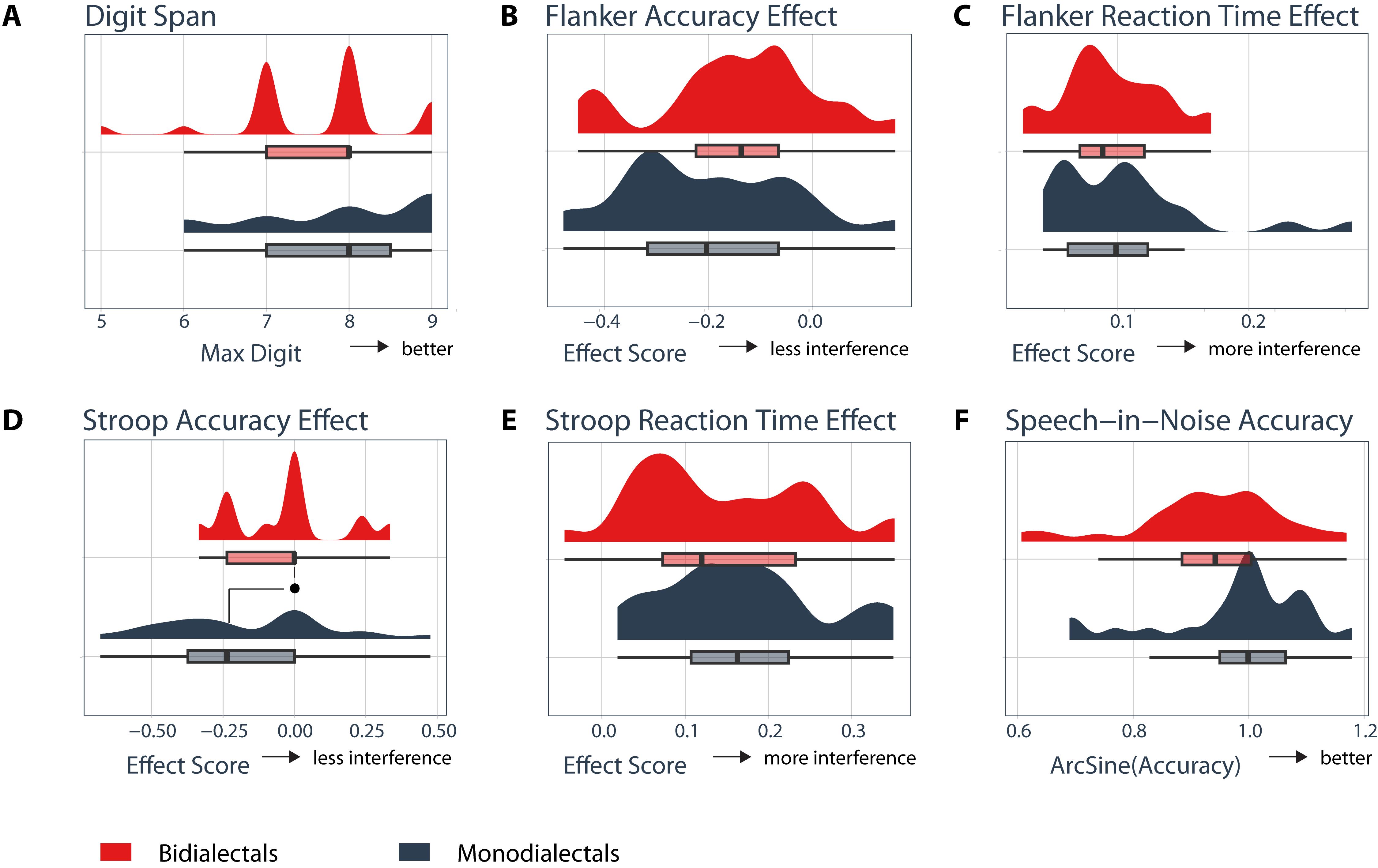


Figure S1: Overview of cognitive test performance of bidialectals (red) and monodialectals (black). A. Raincloud distribution plot for the digit span performance. B. Raincloud distribution plot for the Flanker accuracy effect. C. Raincloud distribution plot for the Flanker reaction time effect. D. Raincloud distribution plot for the Stroop accuracy effect. E. Raincloud distribution plot for the Stroop reaction time effect. F. Raincloud distribution plot for the speech-in-noise accuracy. A dot indicates a statistical trend in the difference between the arithmetic means.


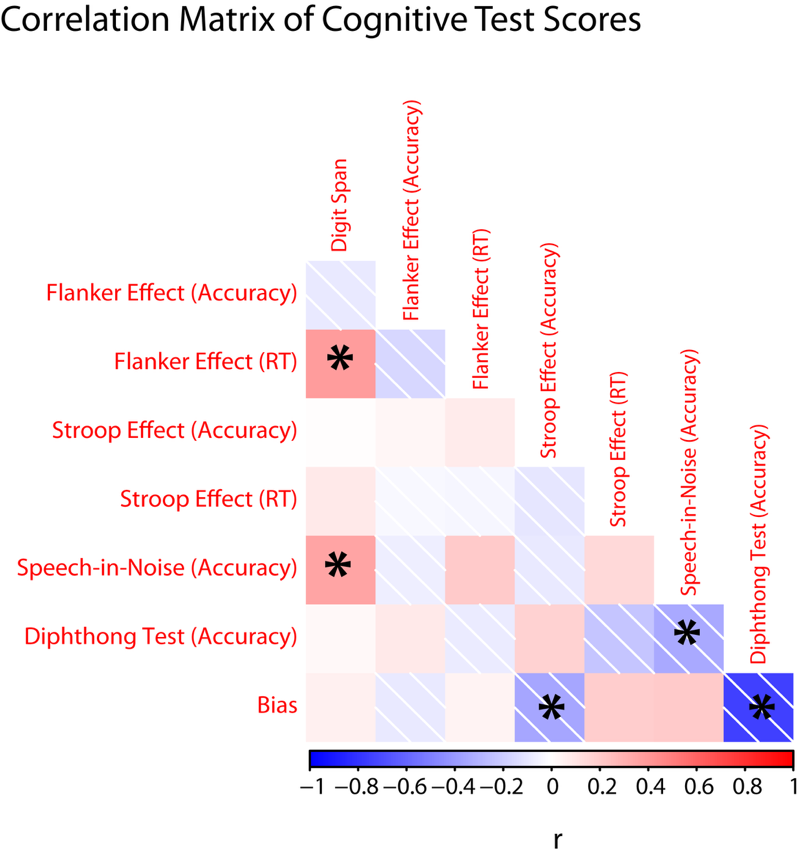


Figure S2: Visualization of the correlation matrix of language and cognitive scores. Asterisks indicate significant Pearson’s correlations.

Table S1: Demographic data of the participants (monodialectals, bidialectals). Abbreviations: AoO – age of onset; aBLP – adjusted bilingual language profile. All participants indicated that English was their 2^nd^ language. Some of the participants indicated that they also spoke a third language. Age of onset of Standard German and Dialect was determined from the aBLP (see Language Tests).

|  | Monodialectals | | | Bidialectals | | |
| --- | --- | --- | --- | --- | --- | --- |
|  | Mean | StdDev | Range | Mean | StdDev | Range |
| % female | 57 |  |  | 54 |  |  |
| % male | 43 |  |  | 46 |  |  |
| Age (years) | 33.65 | 12.39 | 36.00 | 35.69 | 10.90 | 40.00 |
| 2^nd^ language (years) | 11.13 | 8.07 | 34.00 | 10.15 | 4.12 | 18.00 |
| 3^rd^ language (proportion) | 0.74 | 0.45 | 1.00 | 0.54 | 0.51 | 1.00 |
| AoO Standard (years) | 0.09 | 0.29 | 1.00 | 1.08 | 1.98 | 7.00 |
| AoO Dialect (years) | - | - | - | 2.15 | 4.19 | 16.00 |
| Education (years) | 16.04 | 2.20 | 8.00 | 15.04 | 2.46 | 9.00 |
| Bias (aBLP) | 143.01 | 26.38 | 150.86 | 40.31 | 33.50 | 126.51 |

Table S2: Target words for the diphthong test (Standard German). The diphthong goes back to either a monophthong (long vowel) or a diphthong in Middle High German (MHG).

| No | Word form (Standard) | Gloss. | Diphthong (Standard) | Origin (MHG) |
| --- | --- | --- | --- | --- |
| 1 | Eis | ice | [a͡ɪ] | long <i> |
| 2 | beißen | bite | [a͡ɪ] | long <i> |
| 3 | heim | (to) home | [a͡ɪ] | <ei> |
| 4 | Wein | wine | [a͡ɪ] | long <i> |
| 5 | Kleider | cloths | [a͡ɪ] | <ei> |
| 6 | Fleisch | meat | [a͡ɪ] | <ei> |
| 7 | kein | no | [a͡ɪ] | <ei> |
| 8 | weiße | white | [a͡ɪ] | long <i> |
| 9 | Seife | soap | [a͡ɪ] | <ei> |
| 10 | sein | be | [a͡ɪ] | long <i> |

Table S3: Mean, standard deviation and range of cognitive measures for monodialectal and bidialectals.

|  | **Monodialectal** | | | **Bidialectals** | | |
| --- | --- | --- | --- | --- | --- | --- |
|  | Mean | StdDev | Range | Mean | StdDev | Range |
| Digit span (max) | 7.70 | 1.06 | 3.00 | 7.62 | 0.94 | 4.00 |
| Flanker effect  (accuracy) | -0.21 | 0.16 | 0.64 | -0.15 | 0.16 | 0.61 |
| Flanker effect  (reaction time) | 0.11 | 0.06 | 0.24 | 0.10 | 0.04 | 0.14 |
| Stroop effect  (accuracy) | -0.17 | 0.29 | 1.16 | -0.03 | 0.19 | 0.67 |
| Stroop effect  (reaction time) | 0.17 | 0.10 | 0.33 | 0.14 | 0.10 | 0.40 |
| Speech-in-Noise (arcsine accuracy) | 0.98 | 0.13 | 0.49 | 0.93 | 0.13 | 0.56 |
| Diphthong-test (arcsine accuracy) | 0.52 | 0.10 | 0.44 | 1.20 | 0.37 | 1.11 |

References Supplementary Information

1 Kirk, N. W., Fiala, L., Scott-Brown, K. C. & Kempe, V. No evidence for reduced Simon cost in elderly bilinguals and bidialectals. *Journal of Cognitive Psychology* **26**, 640, doi:10.1080/20445911.2014.929580 (2014).

2 Oschwald, J., Schättin, A., von Bastian, C. C. & Souza, A. S. Bidialectalism and bilingualism: exploring the role of language similarity as a link between linguistic ability and executive control. *Frontiers in Psychology* **9**, doi:10.3389/fpsyg.2018.01997 (2018).

3 Poarch, G. J., Vanhove, J. & Berthele, R. The effect of bidialectalism on executive function. *International Journal of Bilingualism* **23**, 612–628, doi:10.1177/1367006918763132 (2019).

4 Ross, J. & Melinger, A. Bilingual advantage, bidialectal advantage or neither? Comparing performance across three tests of executive function in middle childhood. *Developmental Science* **20**, e12405, doi:10.1111/desc.12405 (2017).

5 Lad, M., Holmes, E., Chu, A. & Griffiths, T. D. Speech-in-noise detection is related to auditory working memory precision for frequency. *Sci Rep* **10**, 13997, doi:10.1038/s41598-020-70952-9 (2020).

6 Shokuhifar, G., Javanbakht, M., Vahedi, M., Mehrkian, S. & Aghadoost, A. The relationship between speech in noise perception and auditory working memory capacity in monolingual and bilingual adults. *Int. J. Audiol.* **64**, 131–138, doi:10.1080/14992027.2024.2328556 (2025).

7 Tierney, A., Rosen, S. & Dick, F. Speech-in-speech perception, nonverbal selective attention, and musical training. *J. Exp. Psychol. Learn. Mem. Cogn.* **46**, 968–979, doi:10.1037/xlm0000767 (2020).

8 Schmidtke, J. The Bilingual Disadvantage in Speech Understanding in Noise Is Likely a Frequency Effect Related to Reduced Language Exposure. *Frontiers in Psychology* **7**, doi:10.3389/fpsyg.2016.00678 (2016).

9 Erb, J., Henry, M. J., Eisner, F. & Obleser, J. Auditory skills and brain morphology predict individual differences in adaptation to degraded speech. *Neuropsychologia* **50**, 2154–2164 (2012).

10 Kalikow, D. N., Stevens, K. N. & Elliott, L. L. Development of a test of speech intelligibility in noise using sentence materials with controlled word predictability. *J. Acoust. Soc. Am.* **61**, 1337–1351, doi:10.1121/1.381436 (1977).

11 Mathôt, S., Schreij, D. & Theeuwes, J. OpenSesame: An open-source, graphical experiment builder for the social sciences. *Behavior Research Methods* **44**, 314–324 (2012).

12 Laurencelle, L. & Cousineau, D. Analysis of proportions using arcsine transform with any experimental design. *Frontiers in Psychology* **13**, doi:10.3389/fpsyg.2022.1045436 (2023).

13 Eriksen, B. A. & Eriksen, C. W. Effects of noise letters upon the identification of a target letter in a nonsearch task. *Percept. Psychophys.* **16**, 143–149, doi:10.3758/BF03203267 (1974).

14 Servant, M. & Logan, G. D. Dynamics of attentional focusing in the Eriksen flanker task. *Attention, Perception, & Psychophysics* **81**, 2710–2721, doi:10.3758/s13414-019-01796-3 (2019).

15 Scaltritti, M., Peressotti, F. & Miozzo, M. Bilingual advantage and language switch: What's the linkage? *Bilingualism: Language and Cognition* **20**, 80–97, doi:10.1017/S1366728915000565 (2017).

16 Jensen, A. R. & Rohwer, W. D. The stroop color-word test: A review. *Acta Psychologica* **25**, 36–93, doi:10.1016/0001-6918(66)90004-7 (1966).

17 Scarpina, F. & Tagini, S. The Stroop Color and Word Test. *Frontiers in Psychology* **8**, doi:10.3389/fpsyg.2017.00557 (2017).

18 Andoni Dunabeitia, J. *et al.* The inhibitory advantage in bilingual children revisited: myth or reality? *Exp. Psychol.* **61**, 234–251 (2014).

19 Bialystok, E. & Craik, F. I. M. Cognitive and Linguistic Processing in the Bilingual Mind. *Cur. Dir. Psyc. Sci.* **19**, 19–23, doi:10.1177/0963721409358571 (2010).

20 Torres, V. L. *et al.* The Contribution of Bilingualism to Cognitive Functioning and Regional Brain Volume in Normal and Abnormal Aging. *Bilingualism-Language and Cognition* **25**, 337–356 (2022).

21 Wechsler, D. *Wechsler Adult Intelligence Scale, manual*. (Psychological Corp., 1955).

22 Baddeley, A. in *Memory* (eds Alan Baddeley, Michael W. Eysenck, & Michael C. Anderson) 71–112 (Routledge, 2020).

23 Prigatano, G. P. Wechsler memory scale: A selective review of the literature. *J. Clin. Psychol.* **34**, 816–832, doi:10.1002/1097-4679(197810)34:4<816::AID-JCLP2270340402>3.0.CO;2-Q (1978).

24 Reisberg, D., Rappaport, I. & O'Shaughnessy, M. Limits of working memory: The digit digit-span. *J. Exp. Psychol. Learn. Mem. Cogn.* **10**, 203–221, doi:10.1037/0278-7393.10.2.203 (1984).

25 Grundy, J. G. & Timmer, K. Bilingualism and working memory capacity: A comprehensive meta-analysis. *Second Language Research* **33**, 325–340, doi:10.1177/0267658316678286 (2017).
